# The inhibitory effect of mesenchymal stem cell on blood–brain barrier disruption following intracerebral hemorrhage in rats: contribution of TSG-6

**DOI:** 10.1186/s12974-015-0284-x

**Published:** 2015-04-01

**Authors:** Min Chen, Xifeng Li, Xin Zhang, Xuying He, Lingfeng Lai, Yanchao Liu, Guohui Zhu, Wei Li, Hui Li, Qinrui Fang, Zequn Wang, Chuanzhi Duan

**Affiliations:** The National Key Clinic Specialty, The Neurosurgery Institute of Guangdong Province, Guangdong Provincial Key Laboratory on Brain Function Repair and Regeneration, Department of Neurosurgery, Zhujiang Hospital, Southern Medical University, Guangzhou, 510282 China; Department of Neurosurgery, The First People’s Hospital of Foshan and Foshan Hospital of Sun Yat Sen University, Foshan, Guangdong 528000 China

**Keywords:** Mesenchymal stem cell, Intracerebral hemorrhage, Blood–brain barrier, Peroxynitrite, TNF-α stimulated gene/protein 6, Nuclear factor-κB, Inducible nitric oxide synthase

## Abstract

**Background:**

Mesenchymal stem cells (MSCs) are well known having beneficial effects on intracerebral hemorrhage (ICH) in previous studies. The therapeutic mechanisms are mainly to investigate proliferation, differentiation, and immunomodulation. However, few studies have used MSCs to treat blood–brain barrier (BBB) leakage after ICH. The influence of MSCs on the BBB and its related mechanisms were investigated when MSCs were transplanted into rat ICH model in this study.

**Methods:**

Adult male Sprague–Dawley (SD) rats were randomly divided into sham-operated group, PBS-treated (ICH + PBS) group, and MSC-treated (ICH + MSC) group. ICH was induced by injection of IV collagenase into the rats’ brains. MSCs were transplanted intravenously into the rats 2 h after ICH induction in MSC-treated group. The following factors were compared: inflammation, apoptosis, behavioral changes, inducible nitric oxide synthase (iNOS), matrix metalloproteinase 9 (MMP-9), peroxynitrite (ONOO^−^), endothelial integrity, brain edema content, BBB leakage, TNF-α stimulated gene/protein 6 (TSG-6), and nuclear factor-κB (NF-κB) signaling pathway.

**Results:**

In the ICH + MSC group, MSCs decreased the levels of proinflammatory cytokines and apoptosis, downregulated the density of microglia/macrophages and neutrophil infiltration at the ICH site, reduced the levels of iNOS and MMP-9, attenuated ONOO^−^ formation, and increased the levels of zonula occludens-1 (ZO-1) and claudin-5. MSCs also improved the degree of brain edema and BBB leakage. The protective effect of MSCs on the BBB in ICH rats was possibly invoked by increased expression of TSG-6, which may have suppressed activation of the NF-κB signaling pathway. The levels of iNOS and ONOO^−^, which played an important role in BBB disruption, decreased due to the inhibitory effects of TSG-6 on the NF-κB signaling pathway.

**Conclusions:**

Our results demonstrated that intravenous transplantation of MSCs decreased the levels of ONOO^−^ and degree of BBB leakage and improved neurological recovery in a rat ICH model. This strategy may provide a new insight for future therapies that aim to prevent breakdown of the BBB in patients with ICH and eventually offer therapeutic options for ICH.

## Background

Intracerebral hemorrhage (ICH) has high mortality and accounts for 10% to 20% of all strokes [1]. ICH, which occurs when a blood vessel within the brain ruptures, causes the accumulation of blood within the extracellular space. ICH always has the following features: compression of adjacent brain tissue due to hematoma, reduction of cerebral blood flow, disruption of blood–brain barrier (BBB) function, and increased brain edema, which all contribute to neurological deterioration [2,3]. In particular, BBB leakage, which is closely associated with brain edema formation, may cause secondary brain damage in ICH patients and lead to disability or death.

The BBB is mainly formed by endothelial cells with complex tight junctions which are governed by intracellular proteins, zonula occludens (ZO) as well as essential transmembrane proteins including occludin, claudins, and junctional adhesion molecules [4]. The BBB maintains the neural microenvironment by regulating the passage of molecules into and out of the brain and protects the brain against microorganisms and toxins in the blood [5]. Disruption of the BBB is an important pathophysiological change after ICH and contributes to formation of vasogenic brain edema, which plays an important role in secondary neuronal death and neurological dysfunction [6,7].

Peroxynitrite (ONOO^−^), which is formed by the diffusion-controlled reaction between nitric oxide (NO) and superoxide [8], can exert a devastating effect on the BBB in several diseases including ICH. Upregulation of three isoforms of NOS, which are essential for ONOO^−^ formation, may be correlated with BBB disruption [9]. Under some circumstances, microglia and astrocyte in the central nervous system can generate NO radicals from inducible NOS (iNOS) activation [10,11]. NO is produced in large quantities by iNOS and leads to ONOO^−^ formation and is thought to be a damaging radical that is responsible for brain injury [12]. ONOO^−^ can disrupt BBB integrity by several mechanisms such as impairing cellular energy metabolism, inhibiting Na^+^/K^+^-ATPase activity, which lead to cytotoxic brain edema, and activating the matrix metalloproteinases (MMPs), which can compromise BBB integrity [13-16]. In addition, sites of enhanced 3-nitrotyrosine (3-NT), which is a hallmark of ONOO^−^, are co-localized with tight junction proteins such as zonula occludens-1 (ZO-1) and claudin-5, indicate the direct disruptive effect of ONOO^−^ on BBB integrity [9].

Although mesenchymal stem cells (MSCs) have been successfully used for treatment of experimental ICH, to the best of our knowledge, no previous studies have investigated the possible protective effect of MSCs on the BBB after ICH. Previous studies have indicated that transplanted MSCs are recruited to the site of injury and contribute to repair by transdifferentiation [17,18]. However, recent investigations have shown that paracrine signaling is the primary mechanism accounting for the beneficial effects of MSCs in response to injury [19,20]. After intravenous infusion of MSCs, the cells trapped as emboli in the lung are activated to express the anti-inflammatory factor TNF-α stimulated gene/protein 6 (TSG-6) and eventually reduce inflammatory responses and infarct size in mice with myocardial infarction [21]. The potential mechanism by which MSCs exert their therapeutic effect involves TSG-6 and has been reported in traumatic brain injury [20], renal tubular inflammation and fibrosis [22], corneal injury [23], and dendritic cell maturation [24]. However, whether intravenous transplantation of MSCs improves BBB function after disruption and whether the mechanism is, at least in part, related to secretion of TSG-6, which inhibits the nuclear factor-κB (NF-κB) signaling pathway and decreases of ONOO^−^ in ICH, remain unclear.

Therefore, the effects of MSCs on BBB leakage in a rat ICH model and their potential mechanisms of action were investigated in this study.

## Materials and methods

### BMMSC isolation, culture, and identification

The steps of bone marrow mesenchymal stem cell (BMMSC) isolation were prepared as described previously [25]. MSCs were isolated from the bone marrow of the femur and tibia of the 5-week-old male Sprague–Dawley (SD) rats. The femur and tibia from both knees were isolated with sterile forceps and surgical scissors, and both ends of the long bones were cut away. Mononuclear cells were isolated by Ficoll-Hypaque density gradient centrifugation for 20 minutes at 1,500 rpm. The collected mononuclear cells were plated at 1 × 10^6^ cells/25 cm^2^ in culture flasks in 5 ml DMEM/F12 (1:1) with 10% fetal bovine serum. Non-adherent cells were removed from the cultures after incubation. When the cells reached 90% confluence, adherent cells were harvested and expanded. MSCs that had undergone three passages were used in this study. Flow cytometry analysis was used for MSCs identification. The antibodies were as follows: FITC-CD29, PE-CD34, FITC-CD44, FITC-CD45, and PE-CD90 (Becton-Dickinson Biosciences, San Jose, CA, USA).

### Animals and experimental groups

Our animal study and protocol was approved by the Southern Medical University Ethics Committee. All animal procedures were performed to minimize pain or discomfort in accordance with current protocols. Adult male SD rats weighting 250 to 300 g were purchased from the Animal Experiment Center of Southern Medical University (Guangzhou, China). Animals were housed under a 12-h light/dark cycle with free access to food and water. The SD rats were randomly assigned to three experimental groups: sham-operated group, PBS-treated group (ICH + PBS), and MSC-treated group (ICH + MSC).

### Intracerebral hemorrhage animal model

ICH was induced via the stereotaxic intrastriatal injection of collagenase type IV (Sigma-Aldrich, St. Louis, MO, USA) as described previously with modifications [26]. In brief, the rats were anesthetized with 10% chloral hydrate (0.3 ml/100 g, i.p.; Sigma-Aldrich, St. Louis, MO, USA). Rectal temperature was maintained at 37°C throughout the surgical procedure using a heating lamp. Animals were placed in a stereotaxic frame and under aseptic conditions, and an incision was made exposing the bregma. A 10-uL microsyringe was inserted stereotactically through the burr hole and into the right striatum which coordinates are 0.2 mm anterior, 5.8 mm ventral, and 3.0 mm lateral to the bregma. Collagenase type IV (0.5 IU) in 2 μl saline was injected over a period of 5 min. After placement for another 5 min, the microsyringe was slowly removed. The burr hole was sealed with bone wax, and the wound was sutured. The sham-operated rats were treated the same way except that they were administered 2 μl sterile saline into the right striatum.

### MSC transplantation

Two hours after ICH induction, MSCs were administered intravenously into the rats as previously described with slight modification [27]. The jugular vein was exposed and then isolated with blunt dissection. A 250-μl Hamilton syringe attached with a 31-gauge needle (Hamilton, Princeton, NJ, USA) was laid into the lumen and fixed in place. The cells (5 × 10^6^) in 200 μl PBS (Invitrogen, Carlsbad, CA, USA) were delivered over 10 min. Then, the needle was withdrawn carefully and incision was closed. As a comparison, an equal amount of PBS without MSCs was administered via jugular vein to animals in the PBS-treated group.

### TUNEL assay

Terminal deoxynucleotidyl transferase-mediated biotinylated-dUTP nick-end labeling (TUNEL) staining was performed 72 h after ICH as previously described with minor modifications [28], by use of the *in situ* cell death detection kit (Roche, Nutley, NJ, USA) according to the manufacturer’s instruction. The slides were analyzed with fluorescence microscopy (Bx51, Olympus Corporation, Shinjuku-ku, Japan).

### Behavioral testing

Behavioral testing was conducted 24 and 72 h after ICH according to the previous study [29]. Briefly, the modified neurological severity score (mNSS) test includes motor, sensory, reflex, and balance tests. The mNSS test is graded on a scale of 0 to 18, where a total score of 18 points indicates severe neurological deficit and a score of 0 indicates normal performance, 13 to 18 points indicate severe injury, 7 to 12 indicate moderate injury, and 1 to 6 indicate mild injury. The mNSS test was monitored by two investigators and both of whom had been blinded to groups.

### Analysis of brain water content

Brain water content was measured 24 and 72 h after ICH as described earlier [3,30]. The brains of the rats were removed immediately after anesthetization followed by decapitation, and the brain was divided into two hemispheres along the midline, and the cerebellum and brain stem were removed. Two hemispheres were weighed on an electronic analytical balance to obtain wet weights and then dried in an electric oven at 100°C for 24 h to obtain dry weight. The brain water percentage was calculated as the following formula: ([wet weight − dry weight] / wet weight) × 100 (%).

### Immunohistochemistry

For immunohistochemistry, the rats were anesthetized and transcardially perfused with cold PBS and 4% paraformaldehyde at 72 h after ICH. Slides were incubated with primary antibodies: anti-MPO antibody (1:100, Abcam, Cambridge, MA, USA), anti-Iba-1 antibody (1:100, Abcam, Cambridge, MA, USA) at 4°C overnight. Following primary antibody incubation, the slides were incubated in secondary antibody. Finally, the nucleus was counterstained with hematoxylin. Images were observed with the use of a microscope (Bx51, Olympus Corporation, Shinjuku-ku, Japan).

### ELISA

The rats were killed at 1, 3, and 7 days after ICH or sham operation, the brain tissues were obtained, and the following cytokine levels were quantified by enzyme-linked immunosorbent assay (ELISA): IL-1β, IL-6, IL-10, tumor necrosis factor (TNF)-α, interferon (IFN)-γ, and transforming growth factor (TGF)-β1. Photometric measurements were conducted at 450 nm using microplate reader (Bio-Rad, Hercules, MA, USA). In the process of ELISA, commercial ELISA kits (Bio-Rad, Hercules, MA, USA) were used following the manufacturer’s instructions.

### Quantitative analysis of blood–brain barrier permeability

BBB leakage was assessed as previously described with slight modification [31]. The rats received 100 μl of a 5% solution of Evan’s blue (EB) in saline administered intravenously 24 and 72 h following ICH. Two hours after EB injection, cardiac perfusion was performed under deep anesthesia with 200 ml of saline to clear the cerebral circulation of EB. The brain was removed and sliced. The two hemispheres were isolated and mechanically homogenized in 750 μl of N,N-dimethylformamide (DMF). The suspension obtained was kept at room temperature in the dark for 72 h. It was centrifuged at 10,000 × *g* for 25 min and the supernatant was spectrofluorimetrically analyzed (*λ*_ex_ 620 nm, *λ*_em_ 680 nm) to determine EB content.

### Imnunofluorescence analysis

Immunofluorescence was performed at 72 h after ICH as previously described [9,32]. After antigen retrieval by heat treatment, the sections were incubated at 4°C overnight with primary antibodies: anti-iNOS antibody (Abcam, Cambridge, MA, USA), anti-3-Nitrotyrosine antibody (Abcam, Cambridge, MA, USA), anti-ZO-1 antibody (Invitrogen, Carlsbad, CA, USA). It was then incubated with the appropriate fluorescence conjugated secondary antibodies for 1.5 h at room temperature. Nuclei were stained by Hoechst 33258 (Sigma-Aldrich, St. Louis, MO, USA) for 10 min at room temperature. The slices were observed underneath a fluorescence microscope (Bx51, Olympus Corporation, Shinjuku-ku, Japan).

### NF-κB assay in brain

Cytosolic and nuclear extracts were prepared as previously described [33,34] with slight modifications. Briefly, the brain tissues from rats were suspended in extraction buffer A containing 0.2 mM phenylmethanesulfonyl fluoride (PMSF), 0.15 μM pepstatin A, 20 μM leupeptin, and 1 mM sodium orthovanadate, homogenized at the highest setting for 2 min, and centrifuged at 1,000 × *g* for 10 min at 4°C. Supernatants represented the cytosolic fraction. The pellets, containing enriched nuclei, were resuspended in buffer B containing 1% Triton X-100, 150 mM NaCl, 10 mM Tris–HCl, pH 7.4, 1 mM EGTA, 1 mM EDTA, 0.2 mM PMSF, 20 μM leupeptin, and 0.2 mM sodium orthovanadate. After centrifugation for 30 min at 15,000 × *g* at 4°C, the supernatants containing the nuclear protein were stored at −80°C for further analysis. The levels of IκB-α and phospho-NF-κB p65 (serine 536) were quantified in the cytosolic fraction from the brain tissue collected 24 and 72 h after ICH, while NF-κB p65 levels were quantified in the nuclear fraction. The filters were blocked with 1× PBS, 5% (*w*/*v*) nonfat dried milk for 40 min at room temperature and subsequently probed with specific Abs IκB-α (1:1000, Santa Cruz Biotechnology, Santa Cruz, CA, USA), or phospho-NF-κB p65 (serine 536) (1:1000, Cell Signaling Technology, Beverly, MA, USA), or anti-NF-κB p65 (1:1000, Santa Cruz Biotechnology, Santa Cruz, MA, USA) in 1× PBS, 5% *w*/*v* nonfat dried milk, 0.1% Tween-20 (PMT) at 4°C overnight. Membranes were incubated with goat anti-mouse IgG (1:1000, Invitrogen, Carlsbad, CA, USA) or goat anti-rabbit IgG (1:1000, Invitrogen, Carlsbad, CA, USA) secondary antibody for 1 h at room temperature. Immunoblots were detected using an enhanced chemiluminescence (ECL) kit (Thermo Fisher Scientific, Waltham, MA, USA) and GAPDH (1:1000, Cell Signaling Technology, Beverly, MA, USA) was employed as the loading control.

### Total RNA extraction and real-time PCR

Total RNA extraction and real-time PCR of TSG-6 was performed as previously described [20]. Total RNA was extracted from tissues around the lesional sites 24 and 72 h after ICH using Trizol reagent (Invitrogen, Carlsbad, CA, USA). One microgram of total RNA was reverse transcribed to cDNA with High Capacity cDNA Reverse Transcription Kits (Applied Biosystems, Foster City, CA, USA). Gene transcription was detected by real-time PCR in an ABI Prism 7500 sequence detection system (Applied Biosystems, Foster City, CA, USA) using specific primers designed from known sequences. GAPDH (1:1000, Cell Signaling Technology) was used as an endogenous control. Sequence-specific primers for TSG-6 and GADPH were showed as follows:

- TSG-6, 5′-GCAGCTAGAAGCAGCCAGAAAG-3′ (forward primer),
- TSG-6, 5′-TTGTAGCAATAGGCGTCCCACC-3′ (reverse primer);
- GAPDH, 5′-AAGGTGAAGGTCGGAGTCAA-3′ (forward primer),
- GAPDH, 5′-AATGAAGGGGTCATTGATGG-3′ (reverse primer).

### Western blotting analysis

Rats were sacrificed 24 and 72 h after ICH by injecting overdose of chloral hydrate. Total tissue protein was isolated from ipsilateral lesional brain tissues using ice-cold RIPA buffer. Protein concentrations were measured with the BCA Protein Assay Kit (Thermo Fisher Scientific, Waltham, MA, USA). The samples were subjected to SDS-polyacrylamide gel electrophoresis and transferred to a polyvinylidene diflouride (PVDF) filter membrane. The membranes were blocked with 5% nonfat milk and incubated with primary antibody (rabbit polyclonal anti-iNOS 1:800, Abcam, USA; mouse monoclonal anti-3-nitrotyrosine, 1:1000, Abcam, USA; mouse monoclonal anti-ZO-1, 1:200, Invitrogen, USA; rabbit polyclonal anti-Claudin-5, 1:800, Novus, USA; rabbit polyclonal anti-matrix metalloproteinase-9 (MMP-9), 1:250, Abcam, USA; mouse monoclonal anti-TSG-6, 1:800, Santa Cruz Biotechnology, USA) overnight. The blots were incubated with secondary antibodies after washing with Tris-buffered saline. Immunoblots were detected using an enhanced chemiluminescence (ECL) kit (Thermo Fisher Scientific), and GAPDH (1:1000, Cell Signaling Technology) was employed as the loading control.

### Statistical analysis

Data are presented as means ± SD and analyzed by SPSS 13.0 software (SPSS, Chicago, IL, USA). Comparison between groups was assessed by Student’s *t* test or one-way analysis of variance (ANOVA). A *P*-value of <0.05 was considered to indicate a statistically significant result.

## Results

### Isolation and characterization of MSCs

The MSCs used in our study were isolated from SD rats’ bone marrow and were analyzed for cell surface antigens at passage three. The results gained by using flow cytometry showed that MSCs were positive for CD29 (99.52%), CD44 (94.63%), and CD90 (99.65%) and were negative for CD34 (1.61%) and CD45 (0.95%).

### The effect of MSC treatment on the number of TUNEL-positive cells

**Figure 1:**
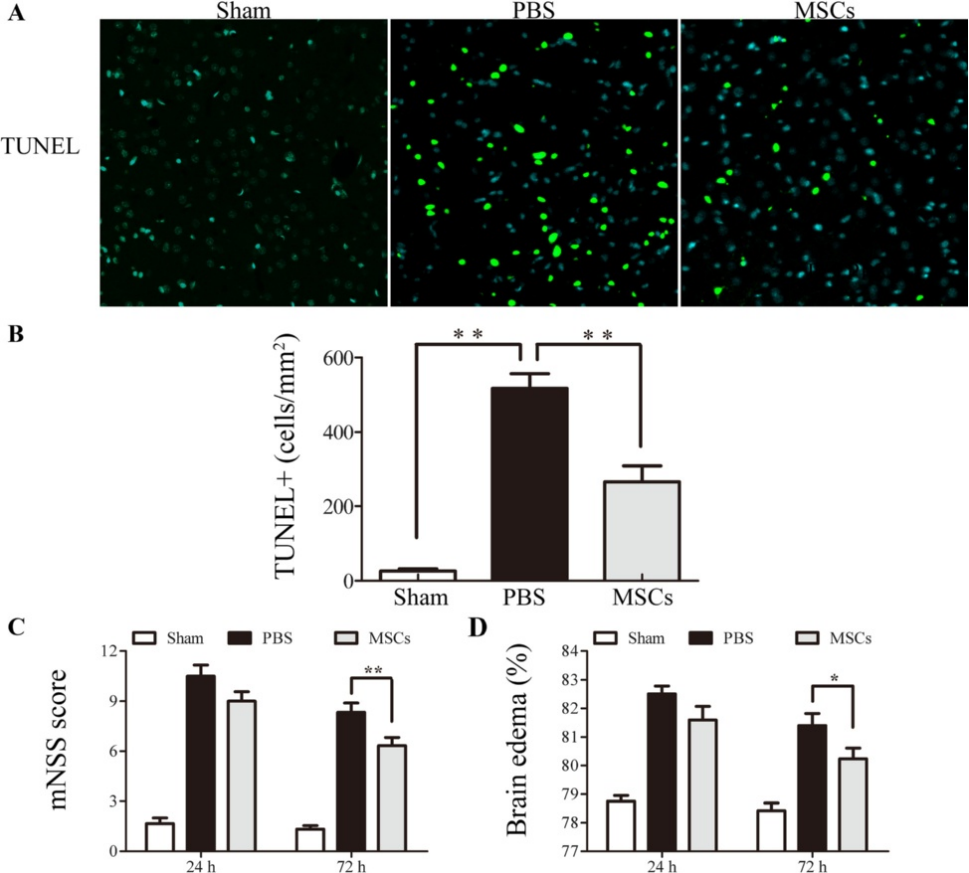
**Effect of MSC transplantation on apoptosis, functional recovery, and brain water content.** Influence of MSC transplantation on apoptosis, mNSS, and brain water content. Compared with the PBS-treated group, the number of TUNEL-positive cells in the cortical hemorrhagic boundary in the MSC group was significantly decreased at 72 h after ICH **(A, B)**. The mNSS and brain water content were tested 24 and 72 h after ICH. Treatment with MSCs significantly lowered mNSS at 24 and 72 h. The mNSS was differed significantly 72 h after ICH between the PBS- and MSC- treated groups **(C)**. The PBS-treated group had a significantly higher brain water content than the sham-operated control group. MSC treatment reduced brain water content compared with the PBS-treated group 24 and 72 h after ICH. The brain water content was different between the two groups 72 h after ICH **(D)**. *n* = 6 in each time point per group. Data are presented as the mean ± SD. *P < 0.05; **P < 0.01. Original magnification, × 600. mNSS, modified neurological severity score; MSCs, mesenchymal stem cells; PBS, phosphate-buffered saline; TUNEL, Terminal deoxynucleotidyl transferase-mediated biotinylated-dUTP nick-end labeling.

In order to investigate apoptotic cells after ICH, TUNEL staining was performed 72 h after ICH (Figure 1A,B). TUNEL-positive cells were detected in the center and the peripheral area of the hemorrhagic lesion. In the sham-operated group, TUNEL-positive cells were barely detected. Compared with the PBS-treated group, the number of TUNEL-positive cells in the cortical hemorrhagic boundary in the MSC-treated group were decreased (*P* < 0.01).

### Improvement of neurological deficits with MSC treatment

We performed the mNSS tests in purpose of examining the effect of MSC transplantation on neurological function. Compared with the PBS-treated group, the improvement in motor performance in the MSC-treated group was statistically significantly different 72 h after ICH (*P* < 0.01) (Figure 1C).

### MSC treatment reduced brain water content

The brain water content was tested to represent the brain edema in hemorrhagic hemispheres and to investigate the effect of MSC treatment on BBB leakage. At 24 and 72 h after ICH, the PBS-treated group had a higher brain water content than the sham-operated group, and brain water content was reduced in the MSC-treated group when compared with the PBS-treated group. The brain water content was statistically significantly different at 72 h between the PBS- and MSC-treated group (*P* < 0.05) (Figure 1D).

### The influence of MSC on brain inflammatory cell infiltration and microglia numbers

**Figure 2:**
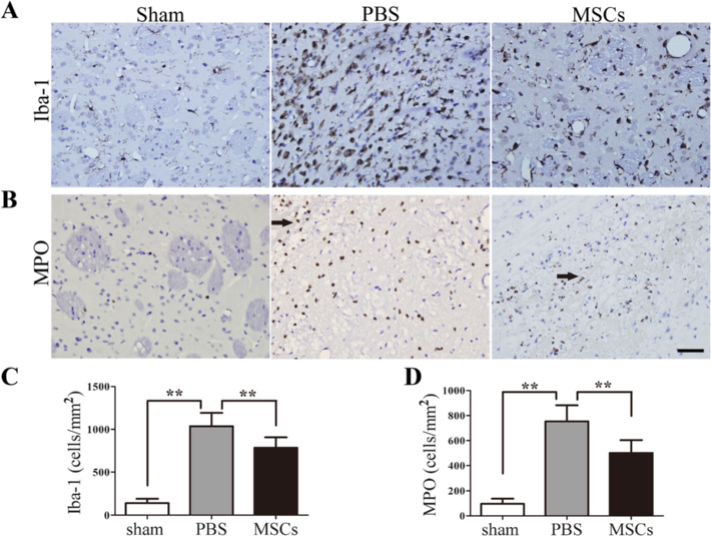
**The influence of MSC on brain inflammatory cell infiltration and microglia numbers.** Iba-1^+^ microglia cells/macrophages and MPO^+^ neutrophils were identified by immunohistochemistry 72 h after ICH to test the effects of MSC treatment on the number of peripheral infiltrating and brain-resident immune cells. Both the numbers of Iba-1^+^ microglia cells/macrophages **(A, C)** and infiltrated MPO^+^ neutrophils **(B, D)** were reduced in the MSC-treated group when compared with the PBS-treated group. The sign of arrow indicates the edge of the hematoma. *n* = 6 per group. Data are presented as the mean ± SD. Bar = 50 μm. **P < 0.01. MPO, myeloperoxidase; MSCs, mesenchymal stem cells; PBS, phosphate-buffered saline.

Iba-1^+^ microglia cells/macrophages and MPO^+^ neutrophils were identified by immunohistochemistry to test the effect of MSC treatment on the number of peripheral infiltrating and brain-resident immune cells. Both the numbers of Iba-1^+^ microglia cells/macrophages (Figure 2A,C) and infiltrated MPO^+^ neutrophils (Figure 2B,D) were reduced in the MSC-treated group when compared with the PBS-treated group (*P* < 0.01).

### Cytokine levels detected by ELISA

**Figure 3:**
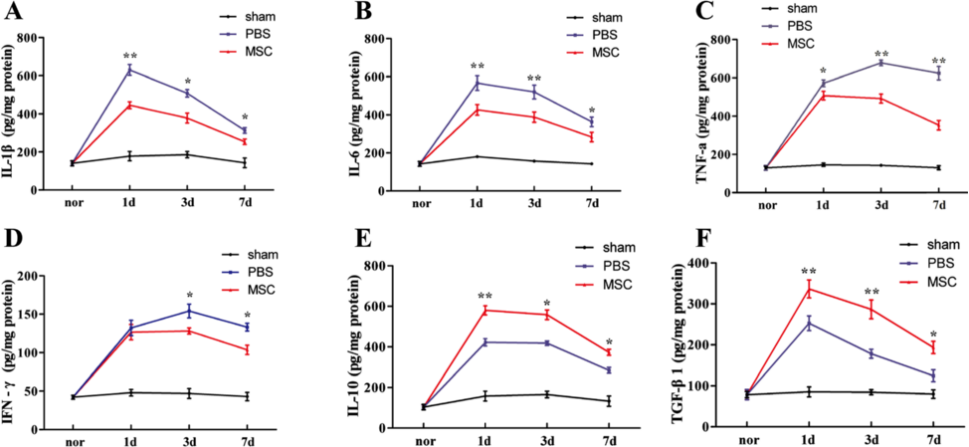
**Influence of MSC treatment on cytokine concentrations.** Levels of the proinflammatory cytokines IL-1β (at 1, 3, and 7 days), IL-6 (at 1, 3, and 7 days), TNF-α (at 1, 3, and 7 days), and IFN-γ (at 3 and 7 days) were decreased in the MSC-treated group compared with the PBS-treated group **(A-D)**. Levels of the anti-inflammatory cytokines IL-10 (at 1, 3, and 7 days) and TGF-β1 (at 1, 3, and 7 days) were increased in the MSC-treated group compared with the PBS-treated group **(E-F)**. *n* = 6 in each time point per group. Data are presented as the mean ± SD. *P < 0.05; **P < 0.01. MSCs, mesenchymal stem cells; PBS, phosphate-buffered saline.

To further assess the microenvironment in the brain which may closely relate to the BBB disruption, we examined the expression of inflammatory-associated cytokines in hemorrhagic lesion at 1, 3, and 7 days after ICH. The levels of IL-1β (at 1, 3, and 7 days), IL-6 (at 1, 3, and 7 days), TNF-a (at 1, 3, and 7 days), and IFN-**γ** (at 3 and 7 days) were all substantially downregulated in the MSC-treated group when compared with the PBS-treated group, whereas the levels of anti-inflammatory cytokines IL-10 (at 1, 3, and 7 days) and TGF-β1 (at 1, 3, and 7 days) were upregulated (*P* < 0.05) (Figure 3).

### Effect of treatment with MSCs on recovery of BBB integrity

**Figure 4:**
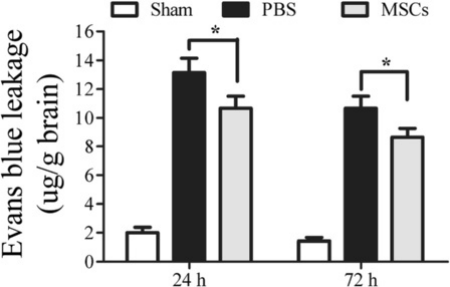
**Influence of MSC treatment on blood–brain barrier permeability.** The intensity of Evan’s blue determined by spectrofluorometry showed that administration of MSCs reduced BBB leakage when compared with the PBS-treated group 24 and 72 h after ICH. *n* = 6 in each time point per group. Data are presented as the mean ± SD. *P < 0.05.

Disruption of the BBB and edema formation is associated with endothelial dysfunction [35]. To investigate the neurovascular protective action of MSCs on ICH, we compared the MSC group to the PBS group in relation to the intensity of Evan’s blue 24 and 72 h after ICH. The intensity of Evan’s blue determined by spectrofluorometric estimation showed that administration of MSCs reduced BBB leakage when compared with the PBS-treated group 24 and 72 h after ICH (*P* < 0.05) (Figure 4).

**Figure 5:**
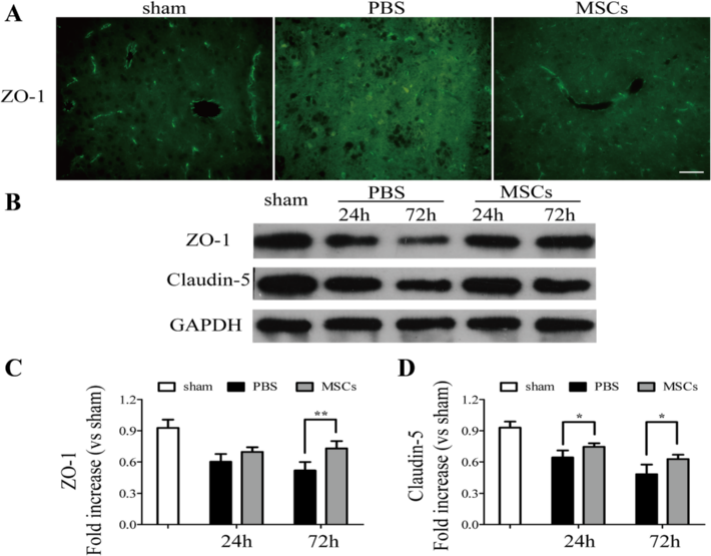
**Transplantation of MSCs increased the levels of zonula occludens-1 (ZO-1) and claudin-5.** Immunofluorescence analysis of ZO-1 and western blotting analysis of ZO-1 and claudin-5. Immunofluorescence analysis of ZO-1 **(A)** showed that treatment of MSCs increased the levels of tight junction protein compared with the PBS-treated group 72 h after ICH. Western blotting analysis of ZO-1 **(B, C)** and claudin-5 **(B, D)** showed similar results in that transplantation of MSCs upregulated the levels of tight junctions compared with the PBS-treated group 24 and 72 h after ICH. *n* = 6 in each time point per group. Data are presented as the mean ± SD. Bar = 50 μm. *P < 0.05; **P < 0.01. GAPDH, glyceraldehyde 3-phosphate dehydrogenase; MSCs, mesenchymal stem cells; PBS, phosphate-buffered saline.

**Figure 6:**
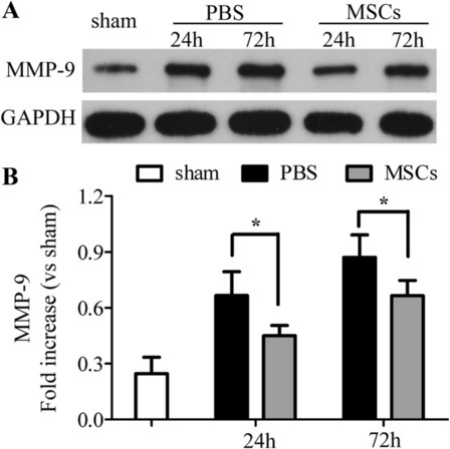
**Transplantation of MSC decreased the levels of matrix metalloproteinase-9 (MMP-9).** Western blotting analysis of MMP-9. Treatment with MSCs downregulated the levels of MMP-9 24 and 72 h after ICH when compared with the PBS-treated group **(A, B)**. *n* = 6 in each time point per group. Data are presented as the mean ± SD. *P < 0.05. GAPDH, glyceraldehyde 3-phosphate dehydrogenase; MMP-9, matrix metalloproteinase 9; MSCs, mesenchymal stem cells; PBS, phosphate-buffered saline.

In addition, tight junction molecules were studied to assess microvascular integrity. As shown in Figure 5, compared with the PBS-treated group, blood vessels in the MSC-treated group were surrounded by more intense and continuous reactivity for ZO-1, which was analyzed by fluorescence microscopy (Figure 5A) and western blotting (Figure 5B,C). Western blotting analysis of claudin-5 (Figure 5B,D) showed similar results in that tight junctions were decreased in the PBS-treated group but increased in the MSC-treated group. The activity of MMP-9, which is also closely related to BBB integrity, was analyzed by western blotting. As shown in Figure 6, transplantation of MSCs reduced the expression of MMP-9, compared with that of the PBS-treated group 24 and 72 h after ICH (Figure 6A, B) (*P* < 0.05).

### The influence of MSCs treatment on the expression of 3-NT and iNOS

**Figure 7:**
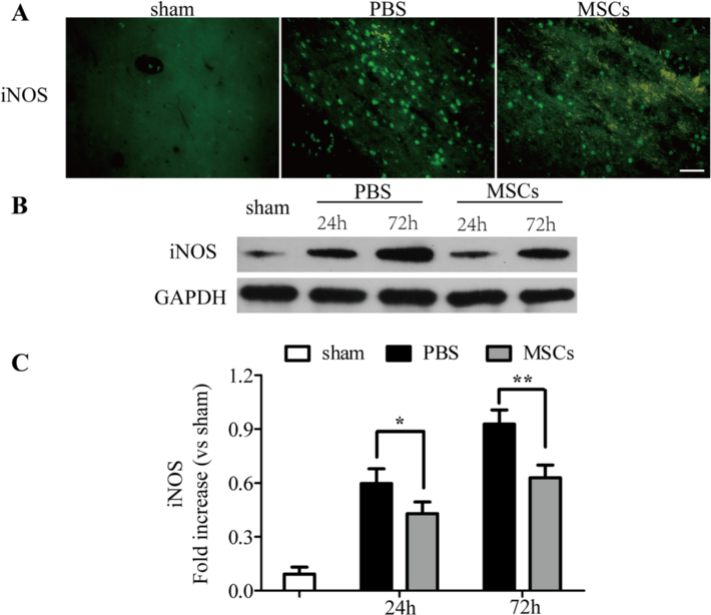
**Transplantation of MSC decreased the levels of inducible nitric oxide synthase (iNOS).** Immunofluorescence and western blotting analysis of iNOS. Immunofluorescence analysis of iNOS **(A)** showed that treatment with MSCs decreased the levels of iNOS compared with the PBS-treated group 72 h after ICH. Western blotting **(B, C)** analysis of iNOS showed similar results in that transplantation of MSCs downregulated the levels of iNOS compared with the PBS-treated group 24 and 72 h after ICH. *n* = 6 in each time point per group. Data are presented as the mean ± SD. Bar = 50 μm. *P < 0.05; **P < 0.01. GAPDH, glyceraldehyde 3-phosphate dehydrogenase; iNOS, inducible nitric oxide synthase; MSCs, mesenchymal stem cells; PBS, phosphate-buffered saline.

**Figure 8:**
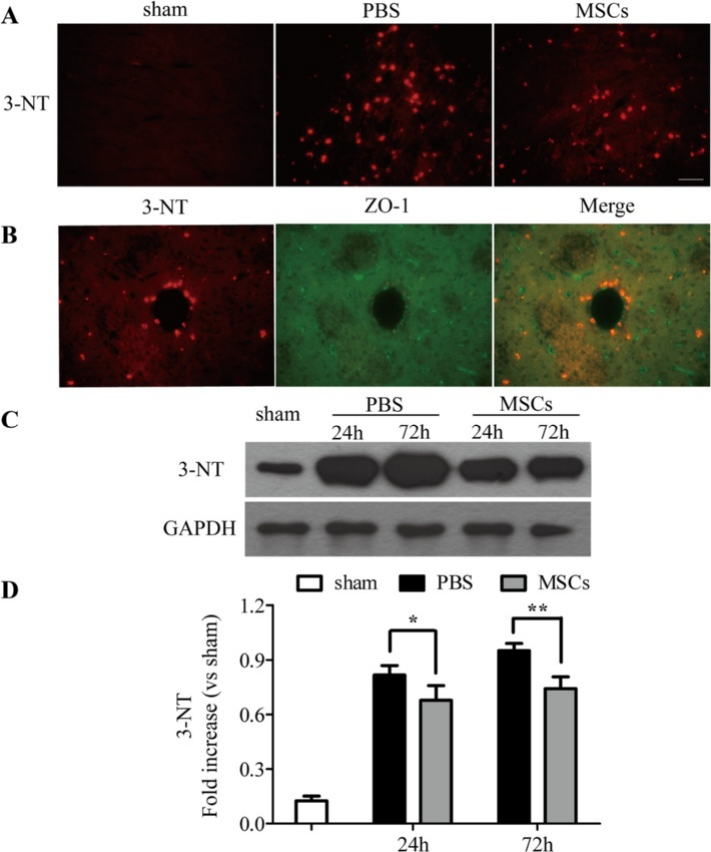
**Transplantation of MSCs decreased the levels of 3-nitrotyrosine (3-NT).** Immunofluorescence and western blotting analysis of 3-NT. Immunofluorescence analysis of 3-NT **(A)** showed that the treatment with MSCs decreased the levels of 3-NT compared with the PBS-treated group 72 h after ICH. Western blotting **(C, D)** analysis of 3-NT showed the similar results in that transplantation of MSCs downregulated the levels of 3-NT compared with the PBS-treated group 24 and 72 h after ICH. The double labeling of 3-NT and ZO-1 indicated that 3-NT and vascular damage are closely related **(B).** *n* = 6 in each time point per group. Data are presented as the mean ± SD. Bar = 50 μm. *P < 0.05; **P < 0.01. GAPDH, glyceraldehyde 3-phosphate dehydrogenase; MSCs, mesenchymal stem cells; PBS, phosphate-buffered saline.

Since ONOO^−^ is unstable, the nitration of tyrosine residues in proteins by ONOO^−^ to 3-NT is a reliable hallmark of the presence of ONOO^−^ [36], so its detection through 3-NT expression is an index of the levels of ONOO^−^ [37]. As shown in Figures 7 and 8, the MSC-treated group decreased the expression of iNOS (Figure 7) and 3-NT (Figure 8A,C,D) obviously when compared with the PBS-treated group. The western blotting analysis of iNOS and 3-NT showed the similar results in that administration of MSCs downregulated the levels of iNOS (Figure 7B,C) and 3-NT (Figure 8C,D) 24 and 72 h after ICH (*P* < 0.05).

### The influence of MSC treatment on the expression of TSG-6

**Figure 9:**
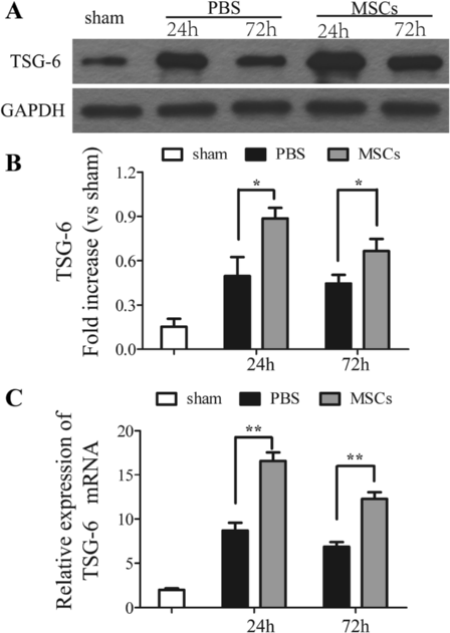
**Influence of MSC treatment on TNF-α stimulated gene/protein 6 (TSG-6).** Western blotting and real-time PCR analysis of TSG-6. Transplantation of MSCs increased the levels of TSG-6 24 and 72 h after ICH compared with the PBS-treated group **(A, B)**. Similar results were observed at the mRNA levels of TSG-6 **(C)**. *n* = 6 in each time point per group. Data are presented as the mean ± SD. *P < 0.05; **P < 0.01. GAPDH, glyceraldehyde 3-phosphate dehydrogenase; MSCs, mesenchymal stem cells; PBS, phosphate-buffered saline; TSG-6, TNF-α stimulated gene/protein 6.

The expression of TSG-6 was detected by western blotting and real-time polymerase chain reaction in order to assess the potential mechanisms which may relate to the protective effect of MSCs on the BBB disruption. As the result of western blotting shown (Figure 9A,B), the treatment of MSC in ICH have upregulated the expression of the inhibitory factors TSG-6 24 and 72 h after ICH (*P* < 0.05); similar results were obtained at the mRNA levels (Figure 9C) (*P* < 0.01).

### Effects of MSC on IκB-α degradation, phosphorylation of Ser536 on p65, expression of NF-κB p65, and NF-κB translocation

**Figure 10:**
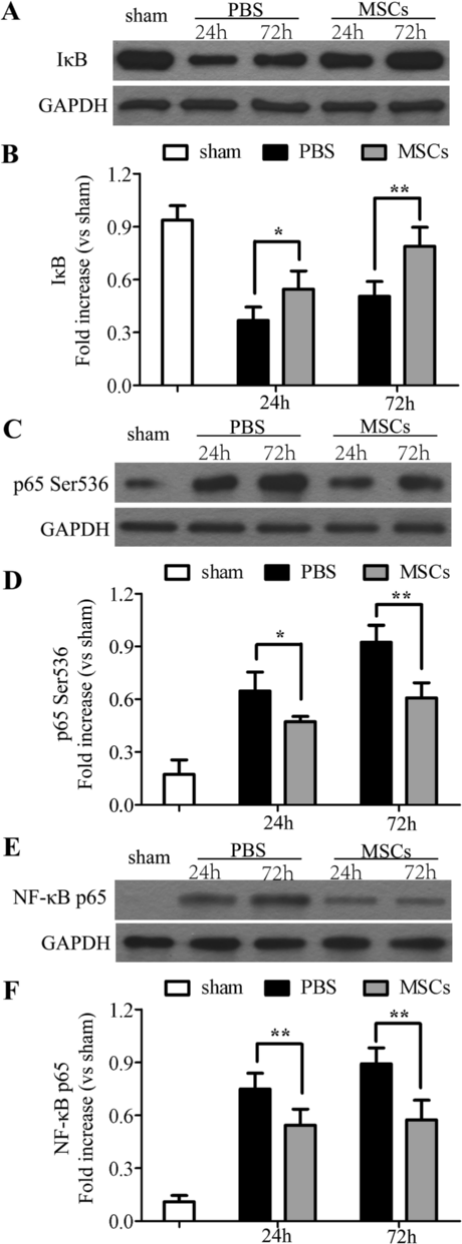
**Effects of MSC on NF-κB signaling pathway.** Treatment with MSCs suppressed activation of the NF-κB signaling pathway 24 and 72 h after ICH. By Western blotting analysis, a basal level of IκB-α **(A, B)** was detected in the brain tissue from sham-operated rats, whereas in the PBS-treated rats, IκB-α levels were substantially reduced. MSC treatment prevented the degradation of IκB-α in the PBS-treated group. Phosphorylation of Ser536 **(C, D)** in the cytoplasm and phosphorylation of NF-κB p65 **(E, F)** levels in nuclear fractions were increased in the PBS-treated group when compared with the sham-operated group. MSC treatment significantly reduced the phosphorylation of p65 on Ser536 and NF-κB p65 levels. *n* = 6 in each time point per group. Data are presented as the mean ± SD. *P < 0.05; **P < 0.01. GAPDH, glyceraldehyde 3-phosphate dehydrogenase; MSCs, mesenchymal stem cells; NF-κB, nuclear factor-кB; PBS, phosphate-buffered saline.

To further investigate the mechanisms by which may attenuate BBB leakage, we evaluated IκB-α degradation, phosphorylation of Ser536 on the NF-κB subunit p65, and total NF-κB p65 by western blotting. Compared with a basal level of IκB-α in the sham-operated group, the levels of IκB-α in the PBS-treated group were reduced; on the contrary, treatment with MSCs inhibited the degradation of IκB-α 24 and 72 h after ICH (*P* < 0.05) (Figure 10A,B). Unlike the levels of IκB-α, phosphorylation of Ser536 on p65 was increased in the PBS-treated group 24 and 72 h post-ICH when compared with the sham-operated group, whereas the treatment of MSC inhibited its increase (*P* < 0.05) (Figure 10C,D). The same as phosphorylation of Ser536 on p65, MSC treatment reduced NF-κB p65 levels in the nuclear fractions of the ICH tissue when compared with the PBS-treated group 24 and 72 h after ICH (*P* < 0.01) (Figure 10E,F).

## Discussion

The main pathophysiological factors of ICH include hematoma size and edema [38]. The formation of edema, which is mainly caused by disruption of the BBB following ICH, is associated with patient outcome. The BBB is composed of endothelial cells, tight junction proteins, astrocyte end-feet, and pericytes, which have the function of maintaining homeostasis of the neuro-parenchymal microenvironment [6]. Loss of BBB integrity is an important pathophysiological change that contributes to initiation of the inflammatory cascade, edema formation, and ultimately poor outcome [39]. In this study, the effect of MSCs on BBB leakage in ICH rats and relevant mechanisms were investigated after intravenous transplantation of MSCs.

Besides endothelial cell activation, vascular ONOO^−^, which is formed by NO and superoxide anion, is closely related to BBB leakage [37]. Studies have already shown that ONOO^−^ alone is sufficient to induce BBB leakage, endothelial dysfunction, and neurodegeneration [40,41]. Several tight junction proteins, such as claudin-5 [42], occludin [43], and ZO-1 [44], are critical determinants of BBB permeability in rats. In the process of BBB damage, the formation of ONOO^−^ may play an important role by reducing the tight junction proteins. In addition to the impact on the tight junction proteins, ONOO^−^-mediated increased expression of MMP-9 is reported to exacerbate BBB leakage [45]. MMPs are important for normal physiological brain function, but in the early stage of ICH, they can be detrimental [7]. Previous studies have established the link between MMP-9 and degradation of tight junction proteins, BBB disruption, inflammation, and tissue injury [46,47]. Our study showed that the increase in ONOO^−^ in ICH rats may have caused harmful effects, such as BBB disruption and brain edema formation. In addition, the levels of tight junction proteins, including ZO-1 and claudin-5, were decreased whereas MMP-9 was increased. MSC treatment restored the reduced expression of BBB integrity proteins such as claudin-5 and ZO-1 and attenuated BBB leakage. Considering that ONOO^−^ formation is closely related to BBB disruption, our results indicated that MSC blocked BBB leakage by suppressing ONOO^−^.

Several studies have elaborated the effect of MSCs on ICH. The potential mechanisms include increase of immature neurons and synaptogenesis [48], enhancement of survival and differentiation of neural cells [49], reduction of inflammatory infiltration, and promotion of angiogenesis [50,51]. However, recent studies indicate that the capacity of MSCs is related to some soluble factors such as interleukin (IL)-10 [52], indoleamine 2,3-dioxygenase [53,54], prostaglandin E2 [52,55], which is a so-called bystander mechanism of MSCs. More recently, TSG-6, one of the anti-inflammatory factors, has attracted increased attention. Although most MSCs which were infused intravenously into the rats are rapidly trapped in the lung, the trapped cells are activated to express amount of TSG-6 [21]. TSG-6 can play a role by inhibiting components in the inflammatory network of proteases [56], suppressing neutrophil migration into the site of inflammation [57], and interacting through the CD44 receptor on resident macrophages to decrease nuclear translocation of the NF-κB complex [58]. Although published literatures do not exclude the possibility that the MSCs trapped in the lung secreted additional factors in addition to TSG-6, an increasing number of results have suggested that the beneficial effect of MSCs are related to the inhibitory effect of TSG-6 on NF-κB [20,24]. Previous studies have indicated that NF-κB is activated as early as 15 min after ICH, reaching a maximum between 1 and 3 days, and remaining elevated for several weeks [59]. NF-κB is normally sequestered in the cytoplasm and bound to regulatory protein IκBs. In response to a wide range of stimuli, IκB is phosphorylated by the enzyme IκB kinase and the result is the release of the NF-κB dimer, which is then free to translocate into the nucleus [60]. Our study showed that in this ICH model, MSC treatment prevented IκB-α degradation and attenuated phosphorylation of Ser536 in the cytoplasm. Likewise, NF-κB p65 levels in the nuclear fraction were also decreased.

The downstream gene products of NF-κB are closely related to NF-κB signal activity. Therefore, the inhibitory effect of MSCs on NF-κB signaling pathway, via TSG-6, may affect formation of the relevant downstream products. Since MSCs can produce some bioactive molecules in addition to TSG-6, we cannot exclude the possibility that other factors augmented the effect of the TSG-6. Among the downstream gene products of NF-κB, iNOS, which plays a significant role in ONOO^−^ formation, may be closely correlated with BBB disruption. In the brain, there is a close relation between iNOS and ONOO^−^ in brain ischemia [61-63], septic animals [64], and Alzheimer’s disease [65]. Our results confirmed the original hypothesis that along with increased levels of TSG-6 and subsequent inhibition of NF-κB activity, the levels of iNOS and ONOO^−^ were clearly decreased.

Besides exacerbation of edema formation, disruption of BBB integrity by ONOO^−^ is a critical event in the initiation of the inflammatory cascade [39]. Our results indicate that MSCs reduce infiltration of microglia cells and neutrophils, increase the levels of anti-inflammatory cytokines, whereas decrease the levels of proinflammatory cytokines, suggesting that they could act also through attenuating the inflammatory response, thus decreasing BBB disruption.

Taken as a whole, we investigated the properties of MSCs in ONOO^−^ formation and BBB protection in a rat ICH model. By transplantation of bone marrow MSCs from the jugular vein, the MSCs were trapped in the lungs and produced a large amount of TSG-6, which acted by suppressing activation of the NF-κB signaling pathway. In ICH, ONOO^−^ is formed around the vessels that attenuate BBB integrity, destroy tight junction proteins, and suppress tissue inhibitor of metalloproteinases-1 (TIMP-1) to initiate a damage cascade. TIMP-1 is one of the naturally occurring inhibitors of MMPs and inhibits multiple MMP activation [66]. INOS, which is regulated by the NF-κB signaling pathway, is of major importance during ONOO^−^ formation. This study focused on the protective effect of MSCs on BBB disruption in a rat ICH model and indicated that the mechanism of MSCs in ICH rats was related to TSG-6, which improved BBB disruption by inhibiting the NF-κB signaling pathway. The levels of iNOS and ONOO^−^ decrease after the inhibitory effect of TSG-6 on the NF-κB signaling pathway. Although there may be also other factors involved in the decrease in iNOS and ONOO^−^, the inhibitory effect of TSG-6 on the NF-κB signaling pathway, at least in part, was a critical contributor to it in ICH rats.

## Conclusions

In summary, our results indicated that MSCs block ONOO^−^-induced BBB disruption in ICH. This strategy may be useful for future therapies targeting prevention of BBB disruption in clinical ICH patients. However, further studies are required to investigate the mechanisms in more detail.

## Abbreviations

3-NT: 3-nitrotyrosine
BBB: Blood–brain barrier
BMMSC: Bone marrow mesenchymal stem cell
ICH: Intracerebral hemorrhage
iNOS: Inducible nitric oxide synthase
MMP-9: Matrix metalloproteinase-9
mNSS: modified neurological severity score
MSC: mesenchymal stem cell
NF-κB: nuclear factor-кB
NO: nitric oxide
ONOO^−^: peroxynitrite
SD: Sprague–Dawley
TIMP-1: tissue inhibitor of metalloproteinases-1
TSG-6: TNF-α stimulated gene/protein 6
ZO-1: zonula occludens-1.
